# Group Differences: The Relationship between Social Media Use and Depression during the Outbreak of COVID-19 in China

**DOI:** 10.3390/ijerph192113941

**Published:** 2022-10-27

**Authors:** Zhenhua Zheng, Wanting Liu, Liu Yang, Ning Sun, Yingchen Lu, Hong Chen

**Affiliations:** 1 College of Communication and Art Design, University of Shanghai for Science and Technology, No. 516, Jungong Road, Shanghai 200093, China; 2 Institute of Local Governance, Yangtze Normal University, Chongqing 408100, China; 3 College of Architecture & Environment, Sichuan University, No. 24 First South Section First Ring Road, Chengdu 610065, China

**Keywords:** elderly health, physical environment, interpersonal environment, social participation, age differences

## Abstract

The outbreak of COVID-19 at the end of 2019 triggered more psychological problems than usual among the public. During this epidemic, the use of social media was very high, and several studies confirmed a positive correlation between social media use and people’s psychological problems. The Chinese government has subsequently implemented a series of policies concerning the social media environment to tackle this “infodemic”. After the containment of the first COVID-19 outbreak, China saw a new wave of COVID-19 cases in Shijiazhuang, Hebei Province in January 2021. How the optimized social media could have impacted public mental health remained to be revealed. Our research data come from an online survey of Chinese residents during the regional epidemic in Shijiazhuang, with a total of 904 valid samples from 18 different provinces in China. The results showed that this new round of outbreaks caused a high incidence of depression (38.9%) among the public. Compared with relatively advantaged groups, disadvantaged groups have a higher depression. Attributed to the optimization of the social media environment, the prevalence of social media use during the epidemic helped to markedly mitigate anxieties from depression. This is particularly demonstrated in vulnerable groups. We found, for the first time, a change in the relationship between social media use and resident depression, and more importantly, a stronger correlation between social media use and depression in relatively disadvantaged groups. Therefore, during the epidemic, actively optimizing the social media environment has a significant and positive effect on the mental health of residents, especially vulnerable groups.

## 1. Introduction

Since the first case was confirmed in December 2019 in Wuhan, China, COVID-19 has spread out across the world. On 11 March 2020, the World Health Organization announced a global pandemic [1]. Its catastrophic consequences are ubiquitous, greatly changing people’s lives, bringing great pressure to people all over the world [2,3] and causing more mental health problems [4,5,6,7,8,9]. What is worth noticing is that certain groups have been disproportionately affected in this regard [3], such as women [10,11,12], health workers [13], unemployed [13], and other groups. Care for public mental health and reducing psychological issues such as depression during the epidemic have become key issues of study for academia, the international community, and governments [14,15].

The epidemic in China has been well contained since March 2020, with its impact on the lives of ordinary Chinese people gradually diminishing. However, a regional outbreak of new cases hit Shijiazhuang, Hebei, China in January 2021. On 3 January, Shijiazhuang initiated wartime controls as a quick response: The city was closed, external and public transportation were all suspended, and approximately 10.25 million people were practicing self-isolation at home. Nucleic acid tests on all residents were conducted three times on 7 January, 12 January, and 20 January. More than 20,000 people were quarantined in designated locations. The Chinese who had barely returned to their normal lives were once again confronted with the tension of the epidemic.

During the first COVID-19 outbreak, social media worked as the main channel for information on the coronavirus disease and provided a window for thorough communication with the outside world [16,17,18]. Therefore, the way in which social media affected the psychology of ordinary people during the epidemic has become a great concern to scholars and governments [19]. Pertinent research all came up with negative conclusions, that is, the more frequently people use social media, the more psychological problems will be incurred [14,15,20,21]. Some scholars have pointed out that the internal reason for this trend is the “infodemic”. With the spread of the global COVID-19 epidemic, a large amount of information related to the epidemic that is difficult to distinguish between true and false has also been generated on the Internet. This makes it difficult for people to find trusted news and reliable guidance. The World Health Organization calls this phenomenon an “infodemic” [22]. An infodemic is more complex than rumor or misinformation. It not only spreads false news and rumors, but also extends to promoting unproven treatments to mislead the public. Stigmatizing information and conspiracy theories in an infodemic can also be demagogic and may lead to racial discrimination, xenophobia, and distrust of government authorities, causing social unrest and destabilizing societies.

The deterioration of the social media environment has been observed since the outbreak. During this period, false information and fake reports about the coronavirus disease flooded social media, causing unfounded fear among many netizens [23,24]. Massive rumors and health misinformation posed a serious threat to public health [25,26], resulting in more emotional problems among ordinary people.

As research conclusions revealed the negative impact of social media on psychological problems during the epidemic, governments and international health organizations have come to a profound insight into the power of the infodemic and have successively called on governments to pay attention to the optimization of the social media environment [27,28]. The United Nations created a dedicated COVID-19 information portal called “Myth Busters” to provide the public with reliable and up-to-date information, debunk and refute false information, and publish reports on a daily basis [29]. Moreover, WHO actively cooperates with industry groups such as search engines and social media platforms and urges them to fight against and filter fake news on their respective platforms. In order to deal with the infodemic, the Chinese government has also taken various measures to combat epidemic-related rumors and fake news: First, the Supreme People’s Court of China issued the “Guiding Opinions on Punishing Fake News” in January 2021 to guide the enforcement of epidemic-related rumors. Local governments, with the support of the Propaganda Department, Health Commission, Public Security Bureau, and other departments, launched anti-rumor campaigns to crack down on local-related fake news. Second, nationwide and all-round network governance measures were launched. The China Internet joint rumor-repudiation platform opened a column for the prevention and control of the COVID-19 epidemic. Major news apps such as Xinhuanet and Tencent.com provided special sections for refuting rumors. Social platforms such as WeChat and Douyin launched a special rectification action against epidemic-related rumors: The rumored accounts will be banned for a limited time or permanently, and fake news will be refuted or deleted in a timely manner. Search engines such as Baidu launched direct search pages related to the epidemic. Medical science websites such as dxy.com launched the COVID-19 epidemic real-time dynamic website, which includes columns such as epidemic maps, rumor refutation, and protection. In addition, governments and authoritative media actively used social media to provide the public with real, positive news and build public confidence in overcoming the pandemic by spreading positive messages through social media.

Most conclusions regarding the correlation between social media and mental health during the COVID-19 pandemic are based on research at the early stage of the epidemic in the first half of 2020. More than a year has passed since, and the current social media environment has already changed to a great extent. Regarding the second outbreak in Shijiazhuang in January 2021, whether the impact of optimized social media on the mental health of Chinese people has altered was yet to be revealed.

In addition, numerous studies have also focused on the heterogeneity of depression that occurred during the pandemic [30]. For example, during this period, the prevalence of depression in women was significantly higher than that in men [11,20,31], and young people under 40 were more susceptible to depression [32,33,34,35,36]. Those with less education tended to display stronger symptoms of depression [11,31,35]. Patterns can be drawn based on the abovementioned: Groups with a higher incidence of depression are often comparatively disadvantaged groups. This implies that during public health emergencies, particular care should be given to vulnerable groups to prevent and alleviate their mental health problems. The impact of social media on public psychology during the COVID-19 pandemic has received due attention, while the heterogeneity of how social media use can affect mental health remains almost unnoted, and how differently it works on the relatively disadvantaged and the relatively advantaged remains unclear.

**Figure 1:**
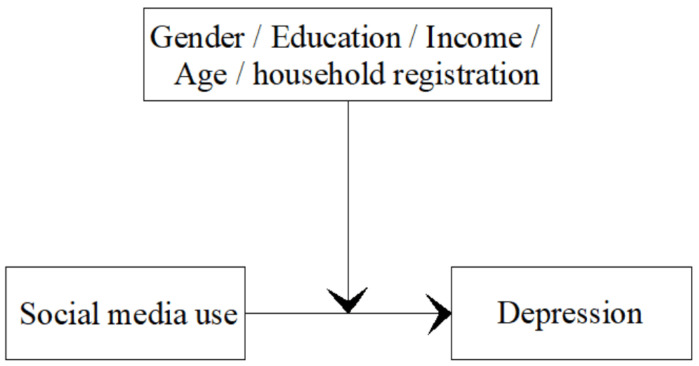
The model of the relationship between social media use and depression in different groups.

In hopes of finding an answer, our research has targeted social media use and depression in ordinary Chinese people during the second regional COVID-19 outbreak in China with a particular emphasis on the vulnerable groups (Figure 1). It aims to obtain reliable information regarding the prevention and alleviation of mental health problems among the vulnerable when a public health emergency occurs. Specifically, the following questions are raised in the research:

1. What was the prevalence rate of depression in ordinary Chinese people during the regional outbreak of the second round of COVID-19 in January 2021? Were relatively disadvantaged groups facing a higher incidence of depression?
2. After the social media environment changed, have there been any changes regarding the impact of social media use on the depression of the Chinese public?
3. How has social media use impacted the depression of different social groups in China? Is there a disparity between relatively disadvantaged and relatively advantaged groups?

## 2. Methodology

### 2.1. Research Design and Participants

This is a cross-sectional study. The data of this study come from an online survey conducted from 10 to 15 January 2021. Shortly after the second round of COVID-19 broke out in Shijiazhuang on 3 January 2021, we launched the survey via Wenjuanxing—an online questionnaire platform (https://www.wjx.cn/app/survey.aspx accessed on 10 January 2021)—to obtain statistics of Chinese residents in a timely manner. Chinese residents over 18 were invited to participate in this online survey, and all participants filled out the questionnaire voluntarily. The survey was conducted under the supervision of the Academic Committee of the University of Shanghai for Science and Technology. Participants were asked to answer specific questions about demographics, social media use, and depression. For each valid questionnaire, the participant would receive a small cash reward (5 RMB per person, approximately 0.78 US dollars). To ensure the validity of the data, we carried out multiple restrictions and multiple screenings in terms of access rights of the questionnaire, the setting of questions, real-name bonus collection, screening of duplicate IPs, and setting the shortest answering time. For example, we interspersed several basic cognitive questions in the question set, including “3 + 6 = ?”, “What is the capital of China?”, “Which picture is a square?”. If the above questions are answered incorrectly, the questionnaire was regarded as invalid. As another example, if one IP, the same device, or the same WeChat account filled in the questionnaire multiple times, we considered the questionnaire invalid. In addition, the questionnaire was invalid if it took less than 150 s to complete. A total of 1204 people from 18 provinces in China took part in the survey. After effectiveness screening, the final valid samples totaled 904 (Table 1).

### 2.2. Measurement

#### 2.2.1. Depression

According to previous studies, depression has always been one of the most prominent mental health problems for the general public, especially when public health emergencies occur [30,31]. It has been found in multiple studies that, after COVID-19 broke out, there had been a high incidence of depression in China [21,23]. In this research, depression was evaluated according to the Chinese version of the WHO-Five Well-Being Index (WHO-5) [37], which includes five positive emotions. The WHO-5 well-being index, also known as the WHO-5 Psychological State Index scale, is a commonly used clinical depression rating scale. It has high validity and reliability in depression screening [38,39,40,41,42]. Therefore, our study chose this scale to measure depression. Participants were asked how often they had had these positive emotions since the outbreak: (1) Feeling peaceful and relaxed, (2) feeling happy and comfortable, (3) feeling refreshed when awake and gratified with rest, (4) being able to find fun in daily life, and (5) feeling full of energy and vitality. Each item is scored on a 6-point scale, with 0 = never, 1 = a minor part of the time, 2 = less than half the time, 3 = more than half the time, 4 = most of the time, and 5 = always. A total score of less than 13 points for the five items suggested the presence of depression.

#### 2.2.2. Frequency of Social Media Use

The frequency of social media use refers to how often participants have accessed information about the coronavirus disease through social media since the outbreak [10], including Weibo (Chinese counterpart of Twitter), WeChat, Zhihu (Chinese counterpart of Quora), Toutiao (news app), Tiktok, and other various media networks. The frequency of social media use was measured by a five-point Likert scale: 1 = never, 2 = occasionally, 3 = sometimes, 4 = often, and 5 = always.

#### 2.2.3. Control Variables

In order to increase the robustness of the model, apart from gender and age, personal factors have been added to our model as control variables including socioeconomic status, housing condition, and health [43,44]. Among socioeconomic factors, we chose education and income as two variables. The rationale behind this is that education and income have been found to be related to mental health [43,44]. The income level is obtained by asking participants for their household monthly income per capita, which is a 6-level item: 1 = <1500 RMB, 2 = 1501–3000 RMB, 3 = 3001–5000 RMB, 4 = 5001–8000 RMB, 5 = 8001–12,000 RMB, 6 = >12,000 RMB. Education is a 5-level item: 1 = junior high school and below, 2 = senior high school, technical and vocational school, 3 = junior college, 4 = undergraduate, 5 = master and above. Based on the experience from existing research, for the living conditions factor, we added the number of people living together as a control variable [45]. Finally, self-rated health (1 = very bad, 2 = relatively bad, 3 = average, 4 = relatively good, 5 = very good) was controlled as it has a direct impact on emotional health. The addition of self-rated health to the covariates is of more help to increase the robustness and effectiveness of the model.

### 2.3. Measurement

The relatively disadvantaged group and the relatively advantaged group are concepts in relative terms. This relativity is reflected in their socio-economic status. In other words, low-income groups are in a relatively disadvantaged position, while high-income groups are relatively advantaged. Similarly, groups with low education levels are relatively disadvantaged, whereas groups with high education levels have an upper hand [46]. In this study, the classification of income and education of the relatively advantage group and the relatively disadvantage group was mainly based on the median value of the sample, in order to ensure the balance of the sample size of the two groups. We defined the family per capita monthly income below or equal to 5000 RMB as the low-income group and above 5000 RMB as the high-income group. In the education level, junior college and below was defined as the group with a low education level, and undergraduate college and above were defined as the group with a high education level. Relativity is also seen in gender: Women are relatively disadvantaged while men are relatively advantaged [47]. Considering China’s specific socioeconomic background, this relativity can also be manifested in age and household registration status. Compared with those of 40 and above, young people under 40 have just set off on their careers and their wealth has yet to be accumulated to ensure financial security. Multiple pressures such as job opportunities, working competition, and high housing prices have put them into the relatively disadvantaged group [48]. In addition, household registration is of particular significance to Chinese people. A registered habitant means relatively stable family support, social networks, and contacts in the local area, suggesting prominent advantages in terms of life convenience and economic stability of work. In contrast, a majority of the migrating population without local household registration are migrant workers lacking the corresponding family and social support. Furthermore, the poor stability of work and finance, and quite often long separation from family members, would put them under more stress, making them a relatively disadvantaged group as compared to registered residents. As students are a special group, there are both local and non-local students. In order to compare the difference between the local registered residents and the migrating population more clearly, this group of samples did not include student samples.

Based on the above analysis, our research classified women, people of low income, low education level, people under 40, and the migrating population as relatively disadvantaged groups, while men, people of high income, highly educated groups, people of 40 and above, and the population with local household registration were defined as the relatively advantaged group.

### 2.4. Statistical Analysis

In this study, structural equation modeling (SEM) was used for statistical analysis, and MPLUS software was selected as the analysis tool. The structural equation model (SEM) makes up for the shortcomings of measurement methods, fuzzy set theory, principal component analysis, factor analysis, and time series clustering. It can better explain the relationship between variables [49]. SEM analysis was combined with factor analysis and path analysis by processing a multivariate covariance matrix. SEM analysis has the following two advantages. First, it allows for independent variables containing measurement error. Second, it can deal with the structural relationship between measurement and factors in a model at the same time. Therefore, this paper used SEM analysis to explore the relationship between social media use and the mental health of residents. In this paper, the Partial Least Squares (PLS) method was used to solve the structural equation model.

In order to test the reliability and validity of the sample data, we divided all observed variables into groups of high and low values and performed *t*-tests, all of which yielded significant results. The groups of high and low values were split by quantiles of 27 and 73. The results showed that all variables were good at discrimination. The depression measurement model was subjected to factor confirmatory analysis, and the results suggested the factor loadings of the observed variables were all above 0.6, the squared multiple correlations were all above 0.36 [50], the construct reliability values of the measurement models were all above 0.6, and the average variance of the extraction amount was above 0.5, indicating that all the measurement models have good reliability and validity. The model fit index GFI value (0.961), RMSER (0.054), chi-square degree of freedom ratio (X^2^/DF), AGFI value (0.953), and IFI value (0.942) all met the ideal standard, suggesting a good fit of the model.

Furthermore, our research is based on cross-sectional data. Under limited conditions, in order to explain the relationship between social media and residents’ depression and avoid the influence of the hybrid effect as much as possible, we chose Propensity Score Matching (PSM) for robustness analysis and causality verification.

PSM achieved the random allocation effect by matching the samples of the treatment group and the control group one by one, so as to control the interference of the self-selection mechanism caused by observable variables [51]. By controlling the propensity value, we can ‘approximately’ satisfy the non-confusion assumption in the framework of counterfactual statistics to make causal inferences [52]. The basic process of PSM was according to observable confusion variables, in which PSM estimates the probability of each sample entering the processing group by Logit model and obtains its propensity score. Then the samples that had the closest tendency but belong to two groups were matched one by one. We used the proximity-matching method to obtain a design effect similar to the random test. Finally, the Average Treatment Effect on Treated (ATT) and the significance of social media on residents’ depression were obtained.

## 3. Results

### 3.1. Prevalence of Depression

It was found that 38.9% of the overall sample had depression. The prevalence of depression in different groups is 41.4% for women and 34.4% for men; 40.1% among low incomes and 38.3% among high incomes; 39.4% for low education levels and 38.6% for high education levels; 40.8% for those under 40 and 37.4% for those of 40 and above; and 41.8% of the migrating population and 38.6% of the registered population (see Table 2).

**Figure 2:**
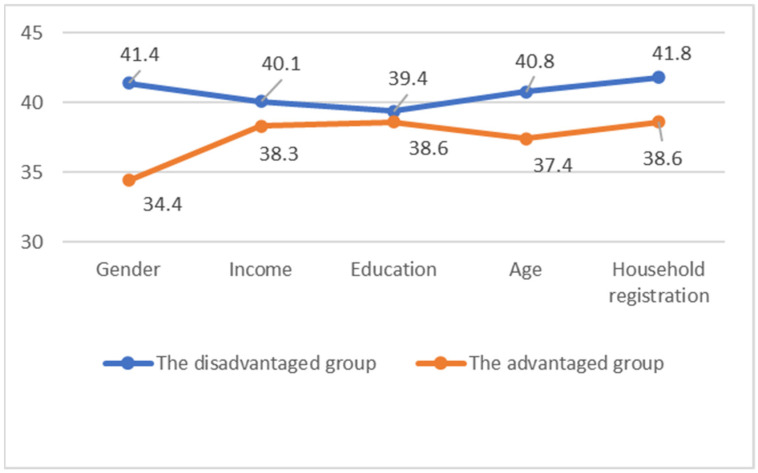
The comparison of the prevalence of depression among relatively disadvantaged and relatively advantaged groups (%).

The results also showed that the prevalence of depression in women is 7% higher than that of men; the prevalence in the low-income group is 1.8% higher than that in the high-income group; the prevalence for the low-education group is 0.8% greater than that of the high-education group; the prevalence of people aged 40 and above with depression is 3.4% more than those under 40; and in the migrating population, this figure is 3.2% higher than that of the registered residents. Among relatively disadvantaged groups (women, low-income, and the migrating population), those suffering from depression all accounted for over 40% (see Table 2 and Figure 2).

### 3.2. The Use of Social Media

In the overall sample of social media use frequency, those who “often” and “always” used social media accounted for 40.3% and 39.8%, respectively, with a total percentage of 80.1. The combined percentages for “frequent” and “always” using social media in different groups were 80.2% among females and 80.0% among males; 78.3% for low income and 81.0% for high income; 75.3% for low education and 83.0% for high education; 81.5% for those under 40 and 79.8% for those 40 and above; and 62.1% for the migrating population and 81.7% for the registered residents (see Table 3).

### 3.3. Overall Model

The model-fitting results based on the overall sample are shown in Table 4. When all covariates are controlled, social media has a significant positive correlation with depression, with a standardized effect value of 0.178 Among the control variables, only age and self-rated health have significant positive impacts on depression, with the standardized impact coefficients being 0.119 and 0.273, respectively.

We used PSM to conduct a robustness analysis and sensitivity analyses on the results of the structural equation model analysis. First of all, we divided the resident sample into an experimental group and a control group according to the difference in social media use frequency. We took the samples with the top 50% use frequency of social media as the experimental group and the remaining samples as the control group. Then we took gender, age, education, income, marital status, number of people living together, years of residence, and SRH as interference factors. We use the binary Logit model to estimate the probability of each sample falling into the experimental group and obtain its propensity score. After that, we matched the samples with the closest propensity score but belonging to two groups, one by one.

To ensure the reliability of the results, we chose the nearest neighbor matching method and the radius matching method. The results showed that the matching success rates of the two matching methods are 100%. The absolute values of standardized deviation after matching were all less than 20%, and the standardized deviation decreased significantly. The *t* tests were not significant (*p* > 0.05), indicating that the matching effect was better.

We performed an average treatment effect analysis for the matched data. (Table 5) The results showed that after the two matching methods were used, the ATT effect was still significant (*p* < 0.05). This showed that after PSM analysis, there were significant differences between social media use and depression, and social media use had a positive effect on depression.

### 3.4. Comparison of Model Paths of Different Groups

Table 6 compares the model fitting results based on samples from different groups. The results show that the frequency of social media use works on depression in significantly different ways across various groups. It has a considerably higher impact on women’s depression than that on men, with respective effect values of 0.218 and 0.111. In low-income groups, its impact on depression is much greater than in high-income groups, with the effect values being 0.251 and 0.129, respectively. The frequency of social media use also works more prominently among low-educated groups compared with those of high education levels, with the effect values being 0.186 and 0.169, respectively. For the group under 40, the impact effect value is 0.247 compared to 0.123 for the group aged 40 and above, almost double the value. The migrant population also saw a more positive effect of social media use on their depression than the local registered population, with impact values of 0.314 and 0.174, respectively.

Among the covariates, only age and self-rated health have a significant impact on female depression; the standardized impact coefficients are 0.174 and 0.274, respectively. Other covariates show no significant correlation with female depression. On the other hand, education, marriage, and self-rated health impact male depression to a great extent, with the standardized impact coefficients being −0.116, −0.137, and 0.251, respectively. For low-income groups, variables such as age, income, and self-rated health impose a prominent effect on their depression, with standardized impact coefficients of 0.186, −0.109, and 0.289, respectively. For high-income groups, education and self-rated health play a crucial role in depression as the standardized impact coefficients are −0.109 and 0.289. In low-income groups, the variables with influence on depression are age and self-rated health, with standardized impact coefficients of 0.177 and 0.272. Self-rated health is the only variable that relates to depression among high-educated groups, with a standardized impact coefficient of 0.281. Gender, marriage, and self-rated health all have a considerable effect on depression in the group under 40; the standardized impact coefficients are −0.104, −0.099, and 0.148, respectively. For those 40 and older, the influential factors are age, marriage, and self-rated health, with standardized impact coefficients of 0.149, 0.092, and 0.383. Gender, education, marriage, and self-rated health have significant effects on depression in the migrant population, and the standardized impact coefficients are −0.298, −0.213, −0.259, and 0.15, respectively. Age and self-rated health contribute to the depression of local registered residents, with standardized impact coefficients of 0.105 and 0.189.

## 4. Discussion

Our cross-sectional research explored social media use and depression in ordinary Chinese people during the second COVID-19 outbreak in China. The purpose of this study is to reveal whether the relationship between social media use and depression changed after the social media environment was optimized in China, and the differences in the relationship among different groups.

Generally speaking, in the case of public health emergencies, the prevalence of mental health problems among ordinary people is higher than that under normal circumstances [53,54,55,56,57,58]. Our research came to the same conclusion. During the second round of the outbreak in China in January 2021, the incidence of depression among Chinese people reached 38.9%, much higher than 6.9% under normal circumstances [52].

We concluded that during the second COVID-19 outbreak in China, vulnerable groups faced more serious problems of depression. During the pandemic, the depression incidence among the public demonstrates obvious heterogeneity characteristics [30,32,33,34], and relatively disadvantaged groups (women, low-income, low-educated, youth, and migrant populations) have a significantly higher prevalence of depression than relatively advantaged groups (male, high-income, high-educated, middle-aged, and locally registered populations).

Similar to previous research conclusions, during the second outbreak, social media was one of the main channels for obtaining information on Coronavirus disease [17,18]. Chinese people are highly dependent on social media with more than 80% of the public using it frequently.

Our research observed a fundamental change in the relationship between social media use and depression in Chinese people during the second COVID-19 outbreak in China. The higher the frequency of social media use, the lower the prevalence of depression. This finding is contrary to the conclusions of previous research [14,15,21]. We believe that an important reason that the impact of social media on depression in the Chinese public has turned from negative to positive may be the optimization of China’s social media environment. After the outbreak of COVID-19 in 2019, a large amount of false news, rumors, and even conspiracy theories about the epidemic spread wildly on social media. This caused public psychological pressure, panic, and emotional problems, resulting in an infodemic. In order to deal with the infodemic, Chinese governments and social organizations enacted various measures to combat rumors and fake news related to the epidemic in social media, and spread positive information through social media to build public confidence in overcoming the epidemic. After a series of governance actions, China’s social media environment has been significantly optimized at the time of the second COVID-19 outbreak. Indeed, social media plays a key role in public mental health, but whether it is positive or negative depends on the quality of the social media environment. A positive and peaceful social media environment is likely to alleviate emotional problems, while a negative and poor environment may exacerbate mental health problems.

More importantly, this study found, for the first time that social media use has a stronger correlation with depression in vulnerable groups. In other words, depression in women, low-income people, low-educated groups, youth, and migrants is much more positively affected by social media. In contrast, men, high-income populations, highly educated groups, and the registered local population benefit less from social media in terms of coping with depression. During the period of a public health emergency, a benign social media environment would allow social media to better mitigate anxieties from the depression of relatively disadvantaged groups. The reason behind this may be that relatively disadvantaged groups are relatively less confident and firm in their belief and are more susceptible to external information [48]. Vulnerable groups normally face greater depression issues; meanwhile, their depression is more likely to be affected by the use of social media. To deal with this problem, not only should governments at all levels and relevant authorities pay special attention and provide assistance to relatively disadvantaged groups, but they should also continue to improve the social media environment, with particular emphasis on increasing care of relatively disadvantaged groups and encouraging groups of women, youth, low incomes, low education level, and migrating populations.

Our research provides government departments and experts with effective evidence to solve the infodemic problem and improve the social media environment from a macro perspective. Moreover, we offer the following suggestions to minimize the public’s mental health issues through the optimization of social media environments. First, the influence of public health and medical professionals on social media should be increased by posting their latest research findings, videos, and health information online, as well as setting up columns, interviews, and popular science sections [59,60] on social media. This is to ensure that the public’s access and understanding are guided by reliable sources and factual information. Second, encouraging the sharing of positive real stories or personal experiences on social media enhances public confidence and helps to improve public misperceptions of COVID-19 and related information [61]. Third, attention should be paid to relatively disadvantaged groups and special groups such as women, low-income, and floating populations. Different groups are affected differently by COVID-19 and social media use [62,63]. Therefore, we need to pay particular attention to those groups with more severe mental health problems during the COVID-19 epidemic and who are more affected by social media. Health information columns, friendly interaction columns, and other service columns dedicated to different groups can be set up on major social media platforms to provide more care and help for these relatively disadvantaged groups.

Additionally, our research also offers advice to the general public on how to deal with infodemic issues from a micro-individual perspective: First, selectively tap into information on social media from trusted sources. In the face of the massive amount of information, correctly identify trusted information as much as possible for dissemination and sharing. Second, people should strengthen independent thinking and critical thinking, with a scientific attitude and rational judgment to evaluate all kinds of information. Individuals should pay attention to distinguish authenticity and not be deceived by false information. Third, individuals should strengthen their sense of social responsibility and responsibility and avoid sharing disinformation, hate speech, and stigmatized information. These will help to improve the mental health of the general public.

## 5. Limitations and Further Direction

Admittedly, some limitations exist in this study. First, our research is based on cross-sectional data. We were only able to explore the relationship between social media use and depression, and further research is needed to follow up. In addition, as the survey is conducted online, there might be certain deviations regarding the representativeness of the sample. For example, participation of the elderly is insufficient, which may affect the evaluation results. However, the advantage of the online survey is that it can obtain data quickly in the case of emergencies. Finally, although we have controlled as many covariates as we could, possible complications incurred by unmeasured factors cannot be ruled out. In further research, we will continue to focus on social media and public mental health issues. In addition, we will focus more on specific measures to minimize public mental health problems through the optimization of social media.

## 6. Conclusions

Our cross-sectional research shows that during the second outbreak of coronavirus in China that occurred in January 2021, Chinese people had more serious depression problems. The depression problems of relatively disadvantaged groups were more serious. This implied that due to the optimization of China’s social media environment, the use of social media may have some moderating effects on depression in the public. This is particularly demonstrated in relatively disadvantaged groups where social media plays a stronger role in coping with depression. More frequent use of social media can help the public, especially relatively disadvantaged groups, to alleviate depression. Therefore, optimizing the social media environment during public health emergencies can help prevent and reduce the mental health problems of the vulnerable. Special attention should be given to increasing the care, guidance, and encouragement for relatively disadvantaged groups of women, low-income, low-education-level, youth, and migrating populations.

## Figures and Tables

**Table 1:** The sample demographics.

Demographics	N	%
**age**		
39−	412	45.58
40+	492	54.42
**gender**		
male	320	35.40
female	584	64.60
**education**		
junior high school and under	58	6.42
senior high school; technical and vocational schools	74	8.19
junior college (with associate degrees)	208	23.01
undergraduate	398	44.03
master and above	166	18.36
**household income per person**		
0–1500 RMB	26	2.88
1500–3000 RMB	100	11.06
3000–5000 RMB	178	19.69
5000–8000 RMB	202	22.35
8000–12,000 RMB	170	18.81
12,000 and above	228	25.22
**marital status**		
unmarried	186	20.58
married	718	79.42
**identity**		
student	110	12.17
local employee	638	70.58
migrant employee	60	6.64
local retiree	82	9.07
family dependent from other cities	14	1.54
**self-rated health**		
excellent	252	27.88
good	458	50.66
fairly good/poor/very bad	194	21.46
**co-habitants**		
1	24	2.65
2	220	24.34
3	372	41.15
4–5	256	28.32
6+	32	3.54

**Table 2:** The comparison of depression prevalence among different groups.

	Depression	
	Number of Sample N	Percentage %
**overall samples**		
**gender**		38.9%
female	242	41.4
male	110	34.4
**average household income (in ten thousand RMB)**		
5000−	122	40.1
5001+	230	38.3
**education**		
junior college and under	134	39.4
undergraduate and above	218	38.6
**age**		
39−	168	40.8
40+	183	37.4
**household registration**		
migrating population	74	41.8
registered residents	278	38.6

Note: This table contains the overall and demographic distributions of the proportion of depression-inflicted people. The total score of all items from the depression scale under 13 indicates the presence of depression.

**Table 3:** The comparison of social media use among different groups.

	Social Media Use					
	Never	Occasionally	Sometimes	Often	Always	Often + Always
**overall samples**	30 (3.3)	68 (7.5)	82 (9.1)	364 (40.3)	360 (39.8)	764 (80.1)
**gender**						
female	16 (2.7)	42 (7.2)	58 (9.9)	230 (39.4)	238 (40.8)	468 (80.2)
male	14 (4.4)	26 (8.1)	24 (7.5)	134 (41.9)	122 (38.1)	256 (80.0)
**household income per person**						
5000−	20 (6.6)	18 (5.9)	28 (9.2)	122 (40.1)	116 (38.2)	138 (78.3)
5001+	10 (1.7)	50 (8.3)	54 (9.0)	242 (40.3)	244 (40.7)	486 (81.0)
**education**						
−junior college and under	22 (6.5)	30 (8.8)	32 (9.4)	134 (39.4)	122 (35.9)	256 (75.3)
+undergraduates and above	8 (1.4)	38 (6.7)	50 (8.9)	230 (40.8)	238 (42.2)	468 (83.0)
**age**						
39−	8 (1.9)	30 (7.3)	38 (9.2)	146 (35.4)	190 (46.1)	336 (81.5)
40+	22 (4.5)	38 (7.7)	44 (5.9)	218 (44.3)	170 (34.6)	388 (78.9)
**household registration**						
migrants without registration	4 (5.4)	8 (10.8)	16 (21.6)	20 (27.0)	26 (35.1)	46 (62.1)
locals with registration	26 (3.6)	50 (6.9)	56 (7.8)	310 (43.1)	278 (38.6)	588 (81.7)

**Table 4:** Standardization coefficient and significance of the overall model.

		Standardization Estimate
**independent variables**	**social media**	**0.178** ***
**covariates**	Gender	−0.021
	Age	0.119 ***
	Education (Edu)	−0.037
	Income (Inc)	0.044
	Marital status (Marri)	−0.017
	Self-rated health (SRH)	0.273 ***
	Co-habitants (Co-ha)	0.010

Note: *** means significant when under 1%.

**Table 5:** ATT effect analysis.

Item		Treated (Experimental Groups)	Control (Control Groups)	Difference (D-Value/ATT Effect Value)	Std. Error	t	*p*
**depression**	Unmatched before matching	3.162	2.696	0.467	0.092	5.074	***
	ATT effect	3.162	2.534	0.628	0.102	6.182	***

Note: *** means significant when under 1%.

**Table 6:** Comparison of path coefficients of different group models.

Variables		Gender		Income		Education		Age		Household Registration	
		Female	Male	Low Income	High Income	Low	High	39−	40+	Migrants	Locals
**independents**	**NMEDIA**	0.218 ***	0.111 **	0.251 ***	0.129 ***	0.186 ***	0.169 ***	0.247 ***	0.123 ***	0.314 ***	0.189 ***
**covariates**	Gender	---	---	−0.026	−0.006	−0.084	0.014	−0.104 **	0.023	−0.298 ***	0.001
	Age	0.174 ***	−0.017	0.186 ***	0.074	0.177 ***	0.065	−0.023	0.149 ***	0.114	0.105 ***
	Education (Edu)	−0.003	−0.116 **	0.067	−0.109 **	0.062	0.018	0.050	−0.070	−0.213 ***	0.019
	Income (Inc)	0.072	−0.042	0.124 **	0.000	0.055	0.027	0.015	0.050	−0.008	0.012
	Co-habitants (Co-ha)	0.009	0.044	−0.008	0.020	0.014	0.010	0.048	0.017	0.004	−0.047
	Marital status (Marri)	0.041	−0.137 **	−0.010	−0.016	0.029	−0.052	−0.099 *	0.092 **	−0.259 **	−0.002
	Self-rated health (SRH)	0.274 ***	0.251 ***	0.252 ***	0.289 ***	0.272 ***	0.281 ***	0.148 ***	0.383 ***	0.157 *	0.297 ***

Note: This table shows the impact of social media use on depression based on different gender, income, education, age, and household registration. *** means significant under 1%, ** means significant under 5%, * means significant under 10%.

## Data Availability

Data are available on request due to privacy or ethical restrictions. The data presented in this study are available on request from the corresponding author.
